# Parameswaran Iyer: transforming sanitation behaviour at scale

**DOI:** 10.2471/BLT.22.030522

**Published:** 2022-05-01

## Abstract

Parameswaran Iyer talks to Gary Humphreys about using community-led behaviour change and frugal technology to transform WASH practices at scale.

*Q: You studied English Literature at St Stephen’s College in Delhi. How did you go from there to working on WASH?*

A: I actually wanted to be a professional tennis player and pursued that goal quite seriously after graduating. But then, when things didn’t work out, I ended up taking and passing the exam for the Indian Administrative Service in 1981 and was assigned as an administrator to the state of Uttar Pradesh where I spent a lot of time working with communities.

*Q: What were you doing?*

A: Everything. As a civil servant in India, you are really thrown into the deep end, covering everything from maintaining law and order to development and infrastructure. It was an incredible experience for me. One of the programmes I got involved in early in my career was something called the Central Rural Sanitation Programme, where I was focused on delivering safe sanitation in rural Uttar Pradesh. Later, I led a World Bank-funded project that is commonly referred to as *Swajal*, the basic idea of which was to empower rural communities to lead the delivery of sustainable health and hygiene benefits to the rural population through improvements in water supply and environmental sanitation services. It was quite a big project, covering about 1000 villages, and it put a big emphasis on strengthening the capacity of rural communities to plan, implement and maintain their water supply and sanitation schemes. It was quite successful and was subsequently picked up by many other programmes in different parts of the world. Community engagement and involvement has since been central to my work with both the Government of India and the World Bank.

“Swachh Bharat is […] taking SDG 6 by the scruff of the neck.”

*Q: You went to work for the World Bank in 1998. What inspired that move?*

A: I had been working for about four and a half years on the *Swajal* project when the Water and Sanitation Programme of the World Bank advertised for a Country Team Leader’s position in Delhi, and I applied for it. I was fortunate to be selected for the job by Piers Cross, the then Regional Team Leader, and an influential global activist in WASH. I relocated to the World Bank in Washington DC in 2000 but resigned in 2006 when I took up the unpaid job of road-manager-cum-coach to my professional-tennis-playing daughter.

*Q: That was quite a change of career path!*

A: It was a wonderful experience. It allowed me to spend time with my daughter and to get back into a sport that had always been a passion of mine. After that interlude, I returned briefly to the civil service, then went back to the World Bank in Washington DC in 2009. I was still working on water and sanitation, but focused on a broad-based water, sanitation, urban portfolio and in different countries, including Egypt, Lebanon and Viet Nam. It was extremely rewarding work, but then, when I saw that India’s newly elected Prime Minister Narendra Modi was prioritizing WASH reform, I felt compelled to go home again. I’ll never forget it. It was 15 August 2014. I was working in Hanoi at the time, and my wife and I were watching the Prime Minister give his first Independence Day address on television. He was giving the address from the ramparts of the Red Fort and he started talking about the need to address the problem of sanitation in India. I was amazed because this was the first time that an Indian Prime Minister had ever spoken about sanitation during a Red Fort speech. About 18 months later, in early 2016, I was fortunate enough to get the opportunity to head up the Ministry of Drinking Water and Sanitation with responsibility for implementing the *Swachh Bharat* Mission.

*Q: Can you tell us a little about the mission?*

A: *Swachh Bharat* is a countrywide WASH campaign which in its first phase was focused on eliminating open defecation. At that time around 550 million people in rural India were defecating outside, having a significant impact on the environment and population health. Empowered by the highest level of political leadership, my job was to try and change that by implementing a behaviour change campaign at scale.

*Q: How did you go about addressing such a monumental challenge?*

A: First by breaking it down to four main challenges – what we called the four Ss: Scale, Speed, Stigma and Sustainability. We were looking at changing the sanitary habits on a huge scale and doing it in five years, which meant building momentum quite quickly and sustaining it. To do so we needed to engage with people at the community level, address the stigma surrounding defecation and provide the infrastructure (toilets) needed to support the transition. And all that more-or-less in parallel and sustainably. On the behaviour change side, we relied on a combination of mass media and community-based influencers, that we call *swachhagrahis*. These were trained to raise awareness of the issue and to get communities to accept responsibility and be accountable. We also solicited the help of several influential celebrities. Our advocacy was greatly supported by the roll-out of a proven safe sanitation mechanism know as a twin pit latrine that is appropriate for use in most parts of the country.

*Q: Can you explain what a twin pit latrine is?*

A: It is a simple, low-cost system that is comprised of a twin compartment leach pit, each compartment lined with honeycomb walls made of brick and an earth floor. The honeycomb allows fluid to leach through while retaining the solid matter. So, the liquid leaches out sideways and below and the aerobic pathogens die, typically over a relatively short period of time. It is also important to mention that the system works with a steep slope toilet pan, which requires less water to flush, so there is less water going into the system in the first place. Typically, for a family of five, it takes about six or seven years for a single compartment to fill up, and when it is full you stop the flow into that pit and divert it to the second pit, allowing the first pit to dry out. Typically, in about a year and a half it dries out and is absolutely safe to handle. You can literally scoop it out with your hands and use it as a fertilizer. There is actually a market for it in India because it's rich in nutrients. I have a plastic bottle of it in my office somewhere and I show it to people all the time.

“People [can] manage their own waste in their own houses.”

The main thing to retain is that, unlike traditional septic tanks, the solid waste does not sit in liquid where it eventually needs to be pumped out and then dumped somewhere else, hopefully at a faecal sludge or water treatment plant. This is a tremendous advantage in rural settings where roads and trucks and treatment plants may be scarce if not altogether absent. So, basically, we promoted the use of this system which was not only cost-effective – it cost roughly the equivalent of US$ 200 to install – but also addressed some of the central concerns regarding proximity to human waste and the stigma that comes with that. We were giving people the means to manage their own waste in their own houses.

*Q: What has been the overall impact of the Swachh Bharat programme?*

A: There have been multiple benefits, including environmental and public health benefits. In terms of the programme itself, a number of external evaluations, including the annual national survey carried out under the World Bank Support Project to the programme, showed that there was a significant rise in access to sanitation, with more than 100 million household toilets being built and a marked improvement in terms of the country’s open defecation free status. That is not to say that there have not been challenges or that there are no gaps in coverage. There are, and the work on sanitation needs to go on, but the overall picture is very positive. But I think the impact goes beyond the programme itself. By being implemented at scale and with such momentum, *Swachh Bharat* is kind of taking SDG [sustainable development goal] 6 [ensure access to water and sanitation for all] by the scruff of the neck and showing what can be done with political leadership, public financing (the federal and state governments committed the equivalent of around US$ 20 billion), partnerships, and people's participation – what we call the four Ps. We have shared the lessons with multiple countries and similar programmes have been set up in Nigeria and Ghana.

*Q: The second phase of the Swachh Bharat programme was approved in 2019 with a deadline to reach its targets for 2024. How will it differ from the first?*

A: The second phase will be devoted to sustaining the outcomes of the first, while broadening the scope of the programme beyond reducing the incidence of open defecation to include solid and liquid waste management in rural areas. Specific targets include organic and plastic waste management and grey water and black water management in rural India.

*Q: You returned to the World Bank in Washington in November of 2021. Do you regret leaving the Swachh Bharat Mission before it is finished?*

A: Implementing the transformational *Swachh Bharat* programme was a phenomenal, once-in-a-lifetime experience and I am very grateful to the Indian government for giving me the opportunity to work on it. I am confident that Phase II of the programme is in safe hands. As for my work with the Bank, I am excited to be working on global projects that address broader issues of water resources and management. Water writ large, so to speak. As programme manager of the 2030 Water Resources Group, an organization housed in the World Bank which works in partnership with the private sector, governments and civil society, I am helping to identify and address major challenges in the water sector, challenges which, with climate change, population growth and displacement and intensification of land use, will only get bigger in the coming years.

